# Tackling Imported Malaria: An Elimination Endgame

**DOI:** 10.4269/ajtmh.14-0256

**Published:** 2015-07-08

**Authors:** Hugh J. W. Sturrock, Kathryn W. Roberts, Jennifer Wegbreit, Colin Ohrt, Roly D. Gosling

**Affiliations:** Malaria Elimination Initiative, Global Health Group, University of California, San Francisco, California

## Abstract

As countries move toward malaria elimination, imported infections become increasingly significant as they often represent the majority of cases, can sustain transmission, cause resurgences, and lead to mortality. Here we review and critique current methods to prevent malaria importation in countries pursuing elimination and explore methods applied in other transmission settings and to other diseases that could be transferred to support malaria elimination. To improve intervention targeting we need a better understanding of the characteristics of populations importing infections and their patterns of migration, improved methods to reliably classify infections as imported or acquired locally, and ensure early and accurate diagnosis. The potential for onward transmission in the most receptive and vulnerable locations can be predicted through high-resolution risk mapping that can help malaria elimination or prevention of reintroduction programs target resources. Cross border and regional initiatives can be highly effective when based on an understanding of human and parasite movement. Ultimately, determining the optimal combinations of approaches to address malaria importation will require an evaluation of their impact, cost effectiveness, and operational feasibility.

## Introduction

Imported malaria infections must be addressed to achieve malaria elimination.^1^ The Global Malaria Eradication Program's failure to eliminate malaria in the 1950s and 1960s underlines the critical nature of this objective, as importation was blamed, in part, for its downfall by reintroducing transmission and spreading chloroquine resistance.^2^,^3^ More recently, importation is thought to have contributed to resurgences of malaria in elimination settings such as Zanzibar as well as countries that have previously achieved elimination such as Greece and Turkmenistan.^4^–^6^ In Swaziland, continued importation of malaria from neighboring Mozambique likely sustains local transmission.^7^ With increasing and more rapid global human movement, preventing the consequences of imported malaria is likely to become a growing issue and developing better methods to reduce the risk of imported malaria will be essential.^8^ Today malaria elimination programs attempt to address importation using a variety of strategies that are poorly defined and not assessed for their effectiveness. Here we review and critique current methods to prevent malaria importation in countries pursuing and maintaining elimination status. While our focus is on elimination settings, we also discuss relevant methods used in other transmission and disease settings and in countries that have successfully eliminated malaria transmission.

### Defining and classifying imported malaria.

The World Health Organization (WHO), the U.S. Centers for Disease Control and Prevention, and most countries define imported malaria as any malaria infection whose origin can be traced to a malarious area outside the country in which the infection was identified.^9^–^11^ Establishing whether an infection was acquired outside the area where it was diagnosed requires knowing the individual's recent travel history. While WHO makes recommendations on the timeframe used to classifying infections as imported, countries use different criteria (Table 1), making it difficult to compare malaria burdens and evaluate strategies to prevent importation.

## Methods to Identify and Prevent Imported Malaria

**Figure 1.**
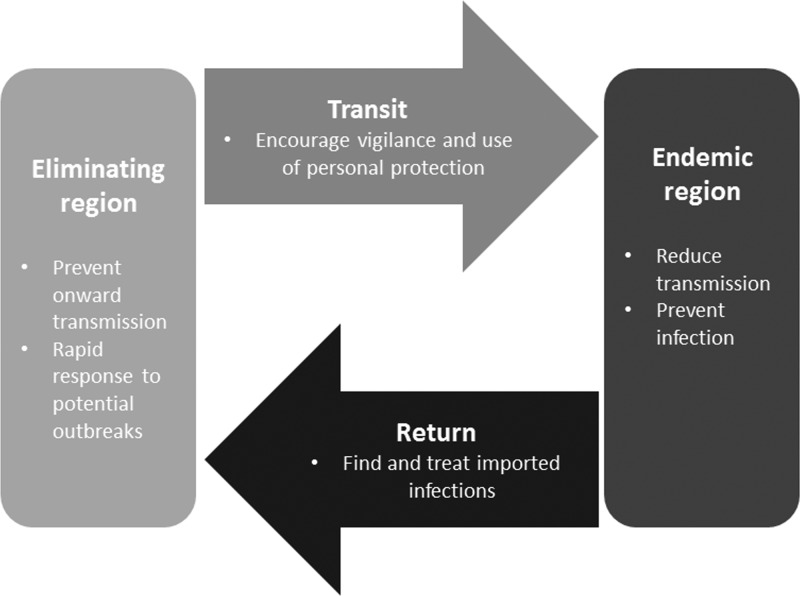
The four stages of human movement and the corresponding objectives of interventions.

From an elimination setting perspective, malaria importation can be addressed during four general stages of human movement: while people are in the eliminating region, during transit, in the endemic region, and upon return to the eliminating country (Figure 1

). Each stage presents an opportunity to prevent acquisition or transmission of imported parasites using a number of interventions (Table 2).

### Improve health infrastructure.

To reduce the contribution imported infections have on local transmission and avoid mortality in returning travelers, countries can improve access to prompt diagnosis and treatment along borders with endemic regions. This can be accomplished by building health facilities at border crossings or along migration routes, an approach implemented in Saudi Arabia and Thailand, and extending free access to noncitizens at existing health facilities, such as access to curative and preventive health services for Angolans in Namibia.^17^–^19^

In low-malaria endemic or receptive malaria-free regions, it is essential to ensure that primary health-care staff members are trained to suspect, correctly diagnose, treat, and rapidly report malaria cases. This important effort requires allocation of resources for continual supervision, training, and monitoring of health providers in both the public and private sectors.

### Border screening.

Voluntary border screening, where individuals are tested and treated at entry points such as airports, ferry terminals, and border posts, is proposed and used as an active surveillance strategy for detecting imported infections.^20^–^22^ Border screening is resource and labor intensive and will miss people using informal border crossings or those with few symptoms. Border screening can be made more efficient by raising the pre-test probability of a positive test through targeting populations such as those coming from known malaria endemic regions or those that have symptoms such as fever, although excluding those without a fever will mean that asymptomatic malaria infections pass without detection.^21^

An important consideration during screening is the field friendliness, cost, and sensitivity of the diagnostic test used. As many infections are likely to be asymptomatic and low density, rapid diagnostic tests (RDTs) and microscopy are likely to miss a large fraction of infections.^23^–^25^ Use of more sensitive, rapid, molecular-based diagnostic tests, such as loop-mediated isothermal amplification (LAMP), could help overcome this, although no test yet exists for detecting the *Plasmodium vivax* dormant liver-stage hypnozoites.^26^ LAMP and polymerase chain reaction (PCR) have not been adopted widely due to novelty, cost (around US$5 per sample), and reliance on a laboratory.^27^,^28^

### Rapid response.

All infections in receptive areas must be rapidly reported to the malaria control program or relevant authority to trigger a timely response to prevent onward transmission and secondary cases. The response may include reactive case detection (RACD), whereby household members and neighbors of index cases in receptive areas are screened for parasites and treated where appropriate,^13^,^16^,^29^ focal indoor residual spraying (IRS)/insecticide-treated bednet (ITN) distribution, larval source management, and education. Focal mass drug administration, a strategy used in China, can be considered as a way to overcome the limited sensitivity of field diagnostics used during RACD.^30^ If such treatment is conducted with long-acting drugs, the prophylactic posttreatment period protects individuals from residual parasites circulating in mosquitoes. If the response system is overwhelmed by numerous cases, prioritization of cases for response can be made based on the likelihood of onward transmission, as is exemplified in Swaziland where prioritization is based on local receptivity derived from risk maps—the most receptive areas being responded to first.^7^

### Provide information, education, and protective measures.

Individuals traveling to or from endemic areas can benefit from information and education about risk avoidance, prophylactic drugs, ITNs, and repellants.^31^ However, while such options may be viable in better-resourced countries, studies show that use of and adherence to chemoprophylaxis can be low irrespective of setting.^32^–^37^

### Reduce receptivity.

Altering local receptivity (the relative abundance of anopheline vectors and the existence of other ecological and climatic factors favoring malaria transmission) may offer a more permanent solution to the threat of importation. While the use of insecticides (IRS, ITNs, and larviciding) is an option, there is a long and successful history of using environmental engineering to reduce malaria receptivity.^38^–^40^ Housing improvements, such as installing window screens and reducing potential breeding sites by removing or covering areas where water collects, reduce receptivity. Housing improvements may be particularly effective when targeted at geographically clustered malaria high-risk populations, such as migrant workers, residing in receptive areas. For example, through legislation and public–private partnerships (PPPs), workers can be housed in low-cost well-screened, or even air-conditioned, housing, a strategy carried out in the United Arab Emirates.^41^ Structural improvements may, however, be costly and deemed unaffordable in many eliminating countries.

### Regionally collaborate to target sources of imported infections.

Perhaps the most effective method of dealing with imported parasites is for networks of countries to target the geographic sources of infections exported to other countries. The Lubombo Spatial Development Initiative (LSDI), a development program between the governments of Mozambique, South Africa, and Swaziland with a large malaria prevention component, demonstrated the value of this approach.^42^,^43^ After ongoing LSDI-supported IRS of insecticides in southern Mozambique between 2000 and 2004, cases in Swaziland and neighboring districts of South Africa decreased by 78–96%.^44^ Another high-profile regional collaboration is the Emergency Response to Artemisinin Resistance in the Greater Mekong Subregion, a WHO-led initiative aimed at coordinating efforts to eliminate malaria in the region.^45^ The Gulf Cooperation Council (GCC) is another example of countries working together to tackle malaria regionally. Through financial and operational collaboration, efforts are targeted at areas of high transmission, such as Yemen, which is a major source of malaria infections in the region.^46^ However, regional collaborations are complex and difficult to sustain and will only be beneficial to elimination countries if an understanding of sources of imported infections are well established and easily targeted with interventions.

## Relevant Identification and Prevention Approaches used in other Disease Settings

### At-source testing and treatment.

Some countries require travelers to have proof of testing or vaccination for tuberculosis (TB), human immunodeficiency virus (HIV), yellow fever, and other infectious diseases before an entry or residence visa is granted.^47^,^48^ Malaria-eliminating countries could require high-risk travelers, such as those arriving from endemic countries, to undergo testing, chemoprophylaxis, or treatment before granting entry or visas. The U.S. Refugee Health Policy includes presumptive predeparture treatment of refugees from sub-Saharan Africa for *P. falciparum* with artemether-lumefantrine.^49^,^50^ While this approach may miss travelers crossing the border informally or without proper documentation, it could be targeted at easy-to-reach high-risk groups, such as military personnel or travelers from highly endemic countries.

### Network sampling.

Network targeting approaches, such as respondent-driven sampling, where sampled individuals help recruit others with similar characteristics, have been used successfully to study hidden and hard-to-reach populations at risk of HIV infection, such as sex workers and injection drug users.^51^,^52^ More recently, these methods have been applied in malaria settings to studies of migrants on the Thai–Cambodia border.^18^,^53^ A recent study in Swaziland, using similar methods, showed that imported cases were able to lead researchers to fellow travelers, potentially at high risk of importing parasites.^54^ Combining network sampling methods with screen and treat or other preventative activities are potentially useful strategies to target imported infections, an approach used for HIV.^55^,^56^ Furthermore, social network research methods may help malaria programs identify times and locations where potential importers gather, facilitating interventions targeted in space and time, and allowing recruitment of highly networked individuals to act as community health workers or volunteers.

### Mobile alerts and reminders.

With the advent of nearly ubiquitous mobile phone technology in most elimination settings, there are many opportunities for mobile disease surveillance and education. Short message service (SMS) alerts directed at individuals moving from endemic to elimination areas may increase the use of ITNs, thereby reducing onward transmission. SMS alerts may also increase the vigilance and treatment-seeking behavior of individuals with symptoms.^57^–^59^ In addition, SMS alerts could be used to notify individuals that they are in a zone where a malaria outbreak is occurring and to take appropriate risk-reduction measures.

## Importation During Prevention of Reintroduction

Once malaria is eliminated, malaria programs are often dismantled and countries have to rely on their broader public health system to ensure that all imported cases are identified, reported, and responded to promptly. This system readiness requires ongoing training of front-line health professionals to ensure that malaria is recognized and diagnosed properly, all malaria cases are promptly reported through the surveillance system, and all cases are investigated to determine whether the infection was acquired locally or abroad. In addition to maintaining vigilance within the public health system, educational campaigns delivered via mass media or targeted at conduits of travel, such as airports, stations, and ferry terminals, can help to ensure those with malaria seek treatment promptly. To monitor how well the passive surveillance system is capturing imported cases, a number of indicators can be used, including the proportion of fever cases tested for malaria to assess testing effort and the proportion of malaria cases that come into contact with the health system within 48 hours of onset of symptoms.^60^

Health system restructuring after elimination may also require other disease control programs, such as dengue or other vector-borne diseases, to conduct field investigations for malaria cases and implement vector control interventions. Ensuring a smooth transition of responsibilities between programs requires careful training, detailed standard operating procedures, and monitoring and evaluation plans.

## Future Directions

Moving forward, there are a number of opportunities to improve the implementation and effectiveness of interventions aimed at tackling imported parasites. First, generating reliable methods to classify infections as imported or local and standardizing those methods across countries would allow accurate comparison between settings and support the evaluation of interventions. Agreeing on standardized approaches based on travel history and establishing parasite genotyping approaches would help address this issue.

Second, there is a need to improve methods for identifying and targeting groups most at risk for importing parasites. A better understanding of who is at risk will make it possible to target high-risk groups at appropriate times and places. Defining groups at high risk of importing parasites can be done using case-control studies, by exploring the social networks of proven cases, and through analysis of routine surveillance data. For routine surveillance data to provide this information, a robust system that is capable of both the collection and analysis of epidemiological data is required. Studies of the movement and malaria-related risk behaviors of undocumented migrants, potentially using network-targeting approaches, are particularly important as little is known about these potentially very high-risk groups.

Third, determining the most appropriate screening tests for the detection of imported infections is an important prerequisite to designing efficient screening programs. Screening for imported infections may be an inefficient use of resources because of the large numbers needed to be screened to detect a rare case and the fact that low-density infections will be missed. If RDTs are not sufficiently sensitive, alternative screening approaches using more sensitive existing molecular diagnostics may be required. Although current molecular detection methods are not field friendly, incentives could be provided to motivate individuals at high risk of malaria infection to travel to a central screening location.

Fourth, there is an opportunity to improve uptake of self-protection measures. The use of protective measures such as chemoprophylaxis, ITNs, insecticide-treated hammocks, or repellents among individuals from elimination areas traveling to higher transmission settings can help travelers prevent infections. However, this approach can be challenging when those that need to be targeted are not well characterized, are missed by outreach efforts, and may not speak the local language. Personal protective measures to address importation are most suitable for well-defined and easy to reach populations, such as military or peacekeeping personnel during deployments or those working in endemic areas. Chemoprophylaxis uptake and adherence can be poor without definitive tests to confirm use. To address such noncompliance in well-organized groups, simple methods to improve medication adherence using directly observed therapy such as double signature checklists or confirmation using mobile devices can be used. Proof of adherence could be required for reentry of these groups into malaria elimination zones. Guidelines and policies that govern how key stakeholders, such as industries employing migrant workers and non-government organizations (NGOs) working with displaced populations, address malaria importation must be in place to ensure appropriate implementation.

Fifth, risk maps can improve targeting of interventions aimed at reducing receptivity. By distinguishing local from imported cases and obtaining information on their location, countries can go beyond coarse risk stratification to generating high temporal and spatial resolution risk maps.^7^,^60^ Target areas can be further prioritized by a consideration of the vulnerability of areas to influx imported infections.^12^ Vulnerability can be crudely estimated by proximity to high transmission area as well as by recording the locations where imported infections reside. More complex estimates of vulnerability can be inferred from human movement data and transmission models, a method used in Zanzibar.^31^

Finally, targeting the sources of imported infections must be prioritized. To address infections across international borders, countries should prioritize the development and growth of regional and cross-border initiatives. Cross-border initiatives, such as the Trans-Kunene Malaria Initiative, show that countries can coordinate efforts, however, funding such strategies can be challenging. One option to fund such work is regional funding pools, resourced by countries affected by importation. Alternatively, cash-on-delivery funding schemes involving many neighboring countries, such as the Global Fund to Fight AIDS, Tuberculosis, and Malaria's Mesoamerica and Hispaniola regional collaboration, are a promising concept that may persuade countries to work together.^61^ It is worth noting that the successful regional collaborations to date, such as the LSDI and the GCC, were designed as initiatives to aid economic development, with malaria control viewed as integral to their success. In addition to funding, regional initiatives require strong political and technical support, which can be provided by initiatives such as the Asia Pacific Leaders Malaria Alliance and the African Leaders Malaria Alliance along with the respective technical partnerships; the Asia Pacific Malaria Elimination Network, the Elimination 8 in southern Africa, and WHO.^1^ Pairing projects that aim to quantify and map human movement patterns with freely available malaria endemicity maps would help identify sources and sinks of parasites. In turn, this can aid the design and implementation of regional approaches aimed at targeting sources of infections.^62^–^64^ Similarly, genotyping parasites, potentially from samples collected through sentinel surveillance sites, would allow further insight into the parasite population structure and movement, a strategy used for Hemagglutinin Type 1 and Neuraminidase Type 1 (H1N1) and HIV.^65^,^66^ While genotyping is recommended by WHO,^67^ standardized methods have yet to be established.

## Conclusions

Imported malaria is a critical obstacle to achieving elimination. There are many ways to address malaria importation and efforts should be tailored to the specific high-risk group importing malaria and the individual country context. Improved strategies to identify and characterize high-risk importation groups will enable targeting and tailoring of interventions. At the local level, efforts should focus on reducing receptivity and preparing for rapid response in receptive and vulnerable areas. At the country and regional levels, collaboration and coordination between countries are essential to allow sources of imported parasites to be targeted. This approach will require a better understanding of where and how parasites move, as well as novel and dedicated regional funding schemes. As an increasing number of countries explore different ways in which to improve their response to the threat of importation, it is important to support and encourage measurement of impact to build the evidence base required to continue shrinking the malaria map.

## Figures and Tables

**Table 1:** Examples of criteria used by eliminating countries to classify cases as imported

Country/organization	Time from visit to endemic country
World Health Organization	3 months^12^
Malaysia	2 months^13^
Sri Lanka	18 days^14^
South Africa, Philippines	1 month^13^,^15^
Swaziland	4 weeks^16^

**Table 2:** Strategies to address importation at different stages of movement

Intervention	Eliminating region	Transit	Endemic region	Return
Improve health-care access	X	X	X	X
Enhance active surveillance	X	X		X
Provide information, education, and communication about prevention	X	X	X	X
Reduce receptivity	X			
Target interventions at sources of infection			X	
Distribution of personal protection	X	X	X	X
Use at-source testing and treatment			X	X
Explore screening incentives	X	X		X
Target networks	X	X		X
Use mobile alerts and reminders	X	X	X	X
